# Nuxcell Neo^®^ improves vaccine efficacy in antibody response

**DOI:** 10.3389/fvets.2024.1248811

**Published:** 2024-02-13

**Authors:** Gabriel Fernandes Alves Jesus, Nathalia Coral Galvani, Jéssica da Silva Abel, Rahisa Scussel, Mírian ĺvens Fagundes, Emily da Silva Córneo, Marina Rossetto, Debora Sargiani, Ricardo Andrez Machado de Ávila, Monique Michels

**Affiliations:** ^1^Biohall Consulting, Research and Innovation, Itajaí, Santa Catarina, Brazil; ^2^Laboratory of Experimental Pathophysiology, UNESC—University of Southern Santa Catarina, Criciúma, Brazil; ^3^Gabbia Biotechnology, Itajaí, Santa Catarina, Brazil; ^4^Biosyn Saúde Animal, Barra Velha, Santa Catarina, Brazil

**Keywords:** vaccine, immunization, probiotic, prebiotic, antibody

## Abstract

Current vaccination protocols raise concerns about the efficacy of immunization. There is evidence that changes in the gut microbiota can impact immune response. The formation of the gut microbiota in newborns plays a crucial role in immunity. Probiotic bacteria and prebiotics present important health-promoting and immunomodulatory properties. Thus, we hypothesize that pro and prebiotic supplementation can improve the efficacy of vaccination in newborns. In this protocol, newborn mice were used and treated with a single-dose rabies vaccine combined with Nuxcell Neo^®^ (2 g/animal/week) for 3 weeks. Samples were collected on days 7, 14, and 21 after vaccination for analysis of cytokines and concentration of circulating antibodies. Our results show an increased concentration of antibodies in animals vaccinated against rabies and simultaneously treated with Nuxcell Neo^®^ on days 14 and 21 when compared to the group receiving only the vaccine. In the cytokine levels analysis, it was possible to observe that there weren't relevant and significant changes between the groups, which demonstrates that the health of the animal remains stable. The results of our study confirm the promising impact of the use of Nuxcell Neo^®^ on the immune response after vaccination.

## Introduction

Vaccination protocols have been an important topic of discussion among veterinarians in recent years, mainly due to concerns about the effectiveness of immunization and its durability. Currently, several types of vaccines are commercially available, but their efficiency is often questioned. Several important questions have typically been addressed when developing vaccines for animals, including whether or not the vaccine is efficacious and cost-effective (1). Immunization is the most important measure to reduce mortality and morbidity from different infectious diseases (2). However, some vaccines are often less effective than expected, as described in a recent review (3). It is known that several factors can interfere with the ideal post-vaccine response, including the age and health condition of the animal, the route of application, malnutrition, compromised immune system, or intestinal dysbiosis (3, 4). There is evidence that dysbiosis can influence vaccine responses (5). The maternal microbiota has been shown to prepare postnatal innate immune development in mice (6). Furthermore, concerning the neonatal immune system, the developing microbiota plays a key role, and dysregulation of the microbiota can lead to a significant impact on systemic immunity (7).

Thus, the intestinal microbiota plays a crucial role in the regulation and development of the immune system; therefore, its composition can affect how individuals respond to vaccines (8, 9). Some results have shown that vaccine effectiveness is low, possibly due to intestinal dysbiosis (8, 9). It is through various mechanisms that the microbiota interacts with the host. It is from microbial colonization during birth that the cross-talks between intestinal bacteria and the host's immune system are initiated (10). This interaction promotes immune homeostasis, the gut epithelial barrier, and protection against pathogenic colonization (11) and inhibits inflammatory reactions (12).

Diet, or nutrition, is one of the main relevant factors in the composition and modulation of the intestinal microbiota. Lack of balance, also known as dysbiosis, leads to different immune disorders (13). Therefore, it is well-reported that the composition of the microbiota affects the effectiveness of interventions related to the immune system, such as vaccination, or even HIV (human immunodeficiency virus infection) (14, 15), cancer immunotherapy (16–18), and different autoimmune diseases (19–25).

By definition, probiotics are living, commensal microorganisms that have positive benefits for the host. Probiotics are generally included in the diet or supplementation and have a positive impact on the immune response, thus reducing infections (26, 27). A recent meta-analysis that included studies with 1,979 adults showed that prebiotics and/or probiotics promoted immunogenicity by influencing seroconversion/seroprotection rates in patients vaccinated against influenza (28). Intervention with probiotic supplements, even during the weaning period of animals, can also demonstrate a more pronounced impact on immune system responses (29). Immunization when performed in newborns is characterized by providing an initial preparation for the immune system, making it more efficient and allowing the possibility of ensuring an excellent basis for future responses (30).

Based on these findings, probiotic and prebiotic supplementation becomes a safe and attractive way to enhance the efficacy of pet vaccines, improving the immune response and enhancing vaccination in newborns through intestinal balance.

## Material and methods

### Animals

In the present study, 15-day-old Swiss mice of both sexes weighing 10–15 g were used. The animals were housed with their parents in a room with a constant temperature of 22 ± 1°C, with water and food *ad libitum*, and subjected to a 12 h light/dark cycle (from 07:00 a.m. to 07:00 p.m.).

Each experimental group consisted of 10 animals and was obtained from the Universidade do Extremo Sul Catarinense (UNESC). No anesthetics were required during the experimental procedure, as it was minimally invasive. No animal died during the experimental protocol. At the end of the study, the animals were euthanized using a lethal intraperitoneal dose of pentobarbital (50 mg/kg).

All methods described were performed in accordance with the relevant guidelines and regulations, including ARRIVE guidelines and the American Veterinary Medical Association (AVMA) Guidelines. All procedures were approved by the UNESC Animal Care and Experimentation Committee (Protocol 16/2022).

### Nuxcell Neo^®^

Nuxcell Neo^®^ was provided by Biosyn Animal Health. Nuxcell Neo^®^ consists of folic acid, nicotinic acid, pantothenic acid, antioxidant additive, arginine, copper, choline, phenylalanine, iron, fructooligosaccharides (FOS), histidine, inositol, iodine, *Lactobacillus casei* CCT 7859, manganese, nucleotides, potassium, *Saccharomyces cerevisiae* ATCC 18824, selenium, zinc, and vitamins A, B1, B12, B2, B6, and D3.

### Experimental design

Initially, the animals were randomly separated into nine distinct groups (10 mice/group): Control (three groups); Vaccine (three groups); and Vaccine + Nuxcell Neo^®^ (three groups), and the euthanasia was performed on days 7, 14, and 21 after vaccination.

The protocol of the rabies vaccine involved a single dose for the vaccine and vaccine + Nuxcell Neo^®^ groups, an inactivated rabies vaccine (Labovet) was administered subcutaneously on the 1st day before treatment.

**Figure 1:**
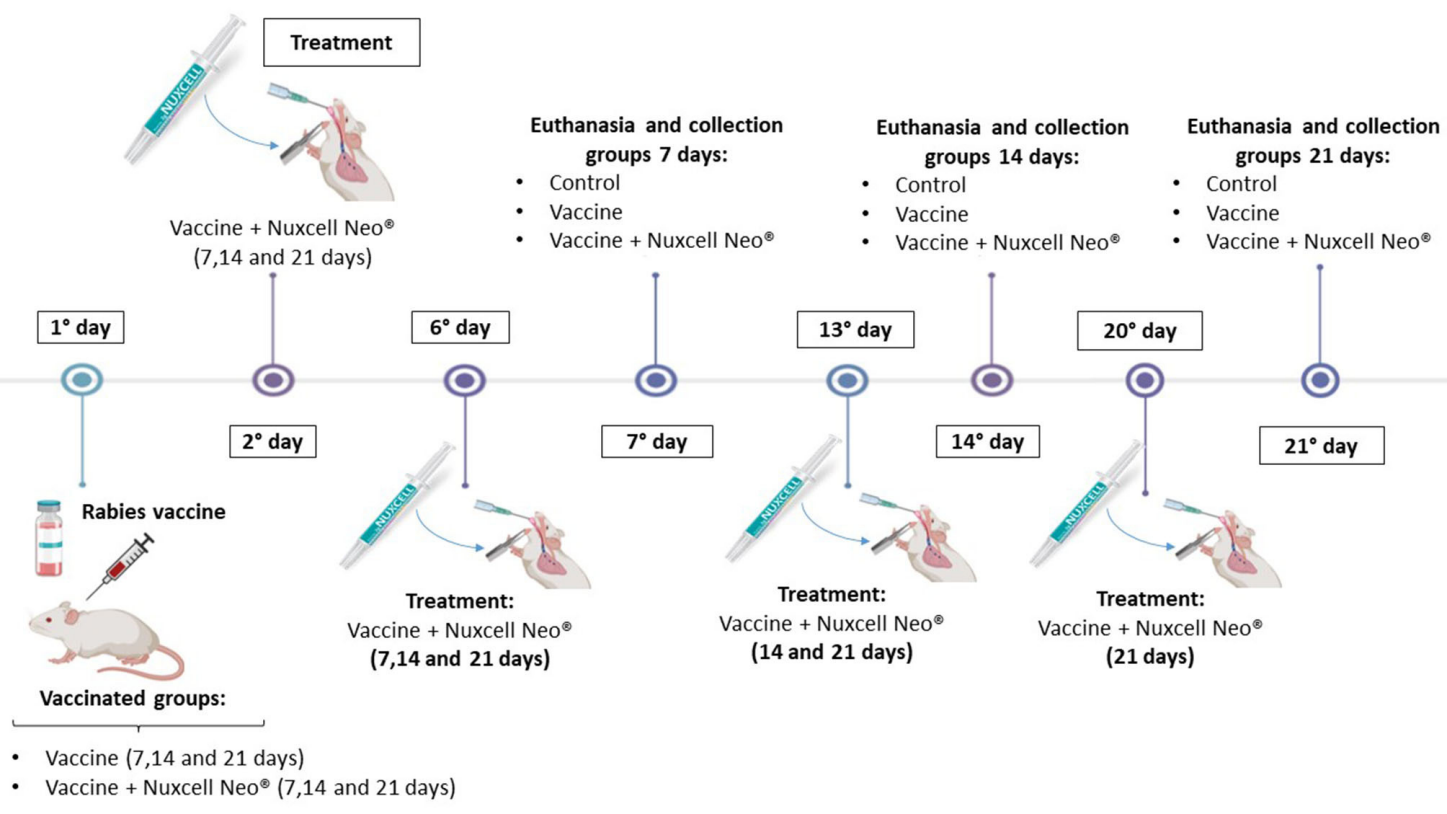
Experimental design.

After the rabies vaccine protocol, treatment with Nuxcell Neo^®^ was started, with a single dose per week (2 g/animal/week), administered on the 6th, 13th, or 20th day via gavage. Serum samples were collected, via the retroorbital route, for analysis, including inflammation [pro and anti-inflammatory cytokines] and the concentration of available circulating antibodies (Figure 1).

### Immunological assays

Titration curves were generated to determine the most appropriate antibody dilution and antigen concentration to be used in the enzyme-linked immunosorbent assay (ELISA). To achieve this, high-binding assay plates (Costar, Corning) were coated with the 25 μl/well inactivated rabies vaccine (Labovet), diluted in coating buffer pH 9.6 (50 mM carbonate buffer), and incubated for 16 h at 4°C. Afterward, the plates were washed twice with phosphate buffer saline (PBS) containing 0.05% Tween 20 (PBS-T) and blocked with 200 μl/well of BSA 5% (Bovine Serum Albumin Protein) with PBST at 37°C for 1 h. After the incubation time, the wells were washed three times, and the serum samples from immunized mice collected throughout the experiment were diluted 1:100 in BSA 0.5% in PBST and then applied at 100 μl per well incubated at 37°C for 1 h. Next, the wells were washed three times with PBST and then incubated with 100 μl/well of anti-mouse-peroxidase antibody (Sigma-Aldrich, Saint Louis, MO, USA), diluted 1:20000 in 0.5% BSA in PBST at 37°C for 1 h. Subsequently, the wells were washed again with PBST. After incubation, colorimetric detection was performed with 0.05% hydrogen peroxide added before pipetting with 100 μl/well. The reaction was incubated for 30 min and stopped with 2M sulfuric acid. The optical density (OD490) was determined by SpectraMax M3 plate spectrophotometer (Molecular Devices, San Jose, CA, USA).

### Cytokine levels

Blood samples were collected and centrifuged at 2800 RCF for 5 min. The concentration of cytokines IL-6 (DY506), IL-1β (DY501), and IL-10 (R1000) was determined by ELISA using a commercial kit (R & D System), and a microplate reader, according to the manufacturer's protocol.

Briefly, high-binding assay plates (Costar, Corning) were coated with capture antibody, diluted in coating buffer pH 9.6 (50 mM carbonate buffer), and incubated for 16 h at 4°C. Afterward, the plates were washed twice with phosphate buffer saline (PBS) containing 0.05% Tween 20 (PBS-T) and blocked with 200 μl/well of BSA 5% (Bovine Serum Albumin Protein) in with PBST at 37°C for 1 h. After the incubation time, the wells were washed three times, and the serum samples from immunized mice collected throughout the experiment were diluted 1:100 in BSA 0.5% in PBST and then applied at 100 μl/well incubated at 37°C for 1 h. Next, the wells were washed three times with PBST and then incubated with 100 μl/well of anti-mouse-peroxidase antibody (Sigma-Aldrich, Saint Louis, MO, USA), diluted 1:20000 in 0.5% BSA in PBST at 37°C for 1 h. Subsequently, the wells were washed again with PBST. After incubation, colorimetric detection was performed with 0.05% hydrogen peroxide added before pipetting with 100 μl/well. The reaction was incubated for 30 min and stopped with 2M sulfuric acid. The optical density (OD490) was determined by SpectraMax M3 plate spectrophotometer (Molecular Devices, San Jose, CA, USA). The unit of measure used was pg/ml.

### Statistical data analyses

All data are expressed as mean ± standard deviation (SD), and all statistical analyses of the data were performed using GraphPad Prism version 5.0 (San Diego, CA, USA). The statistical significance of differences between groups was assessed using one-way ANOVA, followed by *post-hoc* Tukey. Values of *p* < 0.05 were considered statistically significant.

## Results

To assess the effect of Nuxcell Neo^®^ on the vaccination response and its immunomodulatory role, the concentration of serum antibodies after the rabies vaccine was measured and the cytokine levels were analyzed, since the oscillation of pro- and anti-inflammatory cytokines demonstrates the health status of the animals and, in addition, dysbiosis of the intestinal microbiome causes local inflammation.

**Figure 2:**
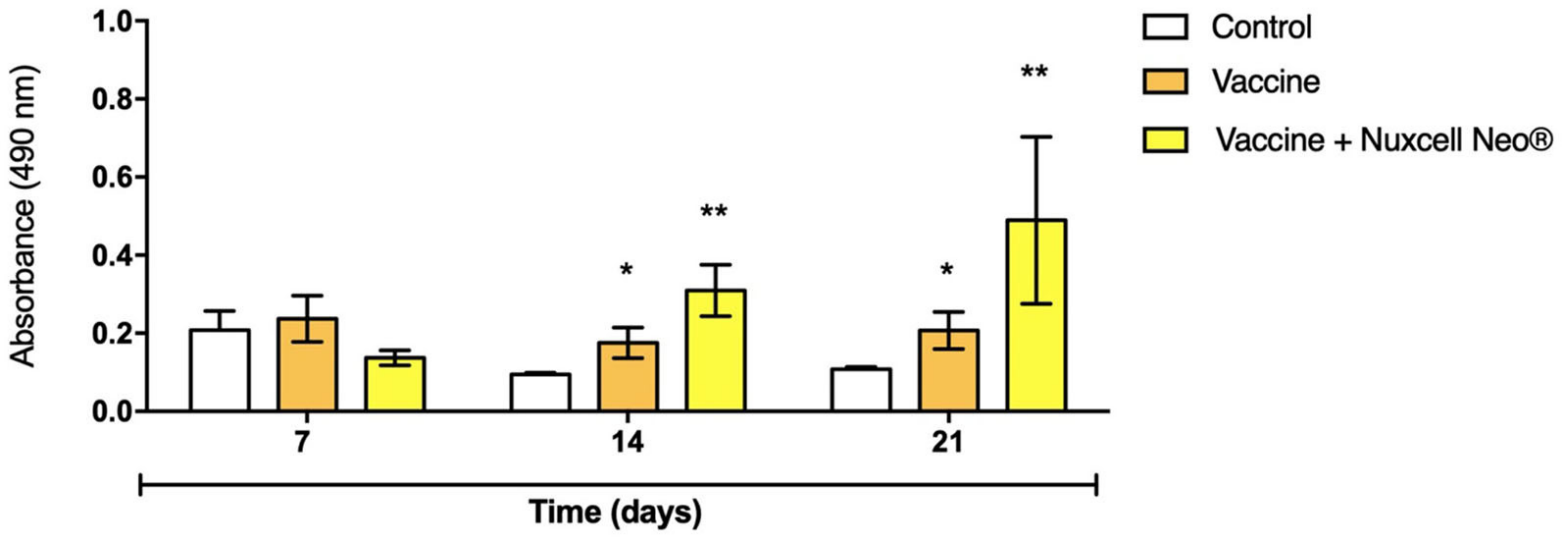
Antibody detection by ELISA assay. Data are expressed as mean + SD, compared to the control group (one-way ANOVA, followed by *post-hoc* Tukey). *n* = 10/group. (*) Significant difference in relation to the control group (*p* = 0.0016); (**) significant difference in relation to the vaccine group (*p* = 0.0017).

Figure 2 shows the result of antibody detection levels after the rabies vaccine and after treatment with Nuxcell Neo^®^. At 7 days, there is no change in levels between the groups (*F* = 1.33; *p* = 0.276), which is expected, since the peak of antibody production is 14 days after vaccination and, therefore, an increase in antibody detection is observed at 14 (*F* = 3.42; *p* = 0.0016) and 21 days (*F* = 4.45; *p* = 0.0017) in the vaccinated group, with an even more significant increase in the group that received supplementation with Nuxcell Neo^®^ when compared to the vaccinated group.

**Figure 3:**
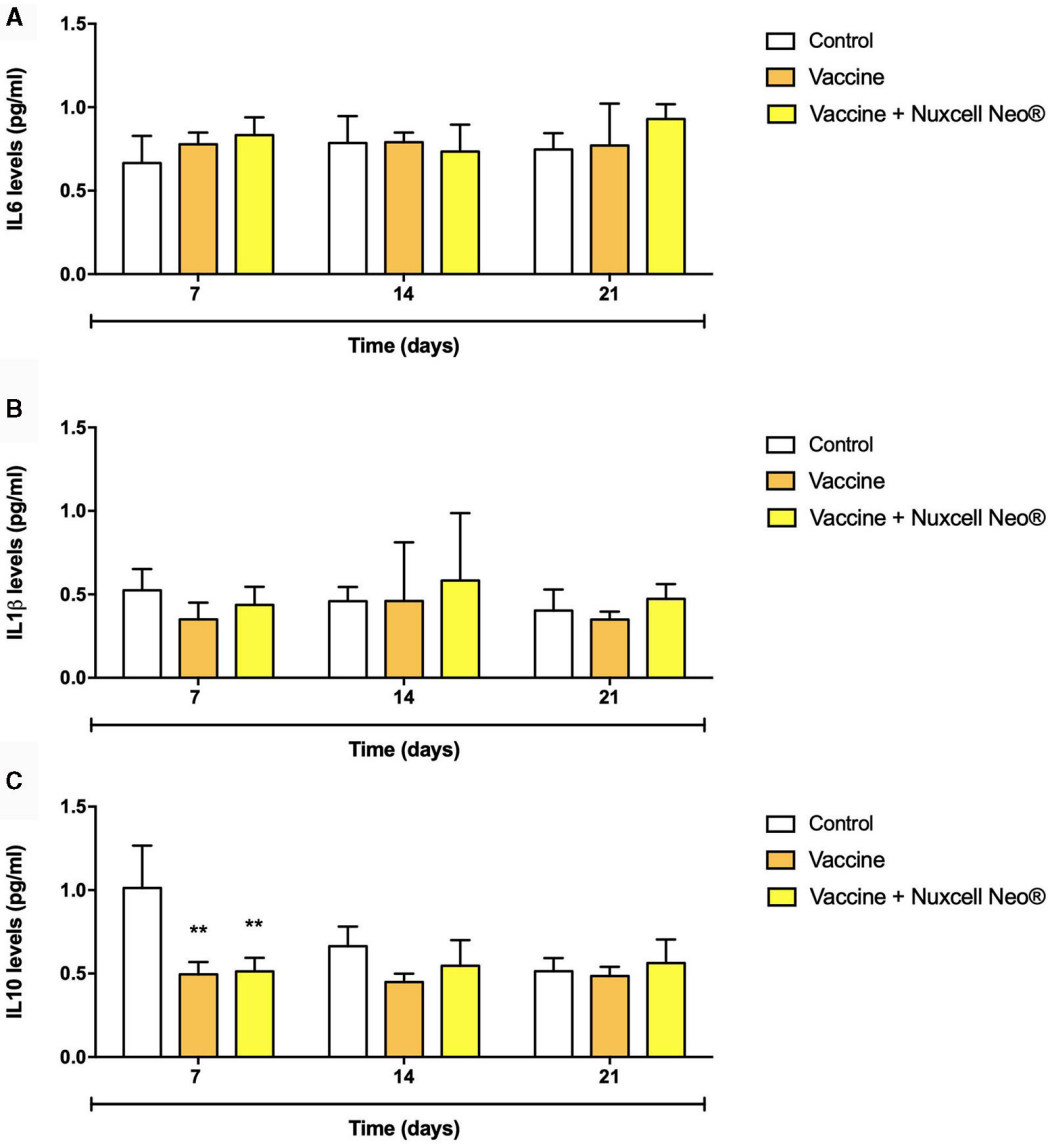
IL-6 **(A)**, IL-1β **(B)**, and IL-10 **(C)** levels were evaluated by enzyme-linked immune absorbent assay (ELISA). *n* = 10/group. Data are presented as mean ± standard deviation (SD), where: **(*p* = 0.03) vs. control group (one-way ANOVA followed by Tukey *post-hoc* test). IL, interleukin.

Figure 3 shows the levels of cytokines assessed. The results show that there are no relevant and significant changes between the groups [IL-6 (*F* = 0.60; *p* = 0.55); IL-1 (*F* = 1.14; *p* = 0.32)], which demonstrates that the animal's health remains stable, without oscillations during the 21 days of follow-up in both groups. This is justifiable since there was no inflammatory insult. Additionally, there was a significant reduction in IL-10 levels at 7 days (*F* = 6.70; *p* = 0.03), but given the normal levels of pro-inflammatory cytokines, this data was not considered detrimental to the study. In addition, there was normalization in the following time points.

## Discussion

The gut microbiota plays a fundamental role in systemic immunity. It is very important in newborns, where the formation of microbiota is essential to prepare the immune system for the high levels of microbes that successively colonize the intestine (12). An imbalance in the intestinal microbiota can cause health disorders and an increase in the incidence of infectious diseases (23). Different aspects of gut development determine the newborn's capacity to tolerate the microbiota, including salts, nutrient, and water transport; and barrier function (13). For this reason, gut microbiota can impact the function and development of vaccine efficacy and humoral immunity (9). The effectiveness of vaccines depends on different factors, one of the most influential being the intestinal microbiota (8). Interactions between gut bacteria and the immune system begin immediately after birth, directly influencing the immune response and, thus, protecting against pathogens (31).

When there are changes in the composition of the gut microbiota, it can lead to several immune disorders, thereby impairing the proper response to immunization (31). Metabolic diseases such as diabetes can alter the gut microbiome and disrupt gut bacterial equilibrium (32). The gut microbiota impacts the effectiveness of various immune system-related interventions, including HIV prevention (15), cancer immunotherapy (16), and dysregulation in gut microbial composition associated with autoantibody production and autoimmune diseases (21). Other factors, including physical activity, mental health, and obesity can also affect the composition of gut microbiota (33).

**Figure 4:**
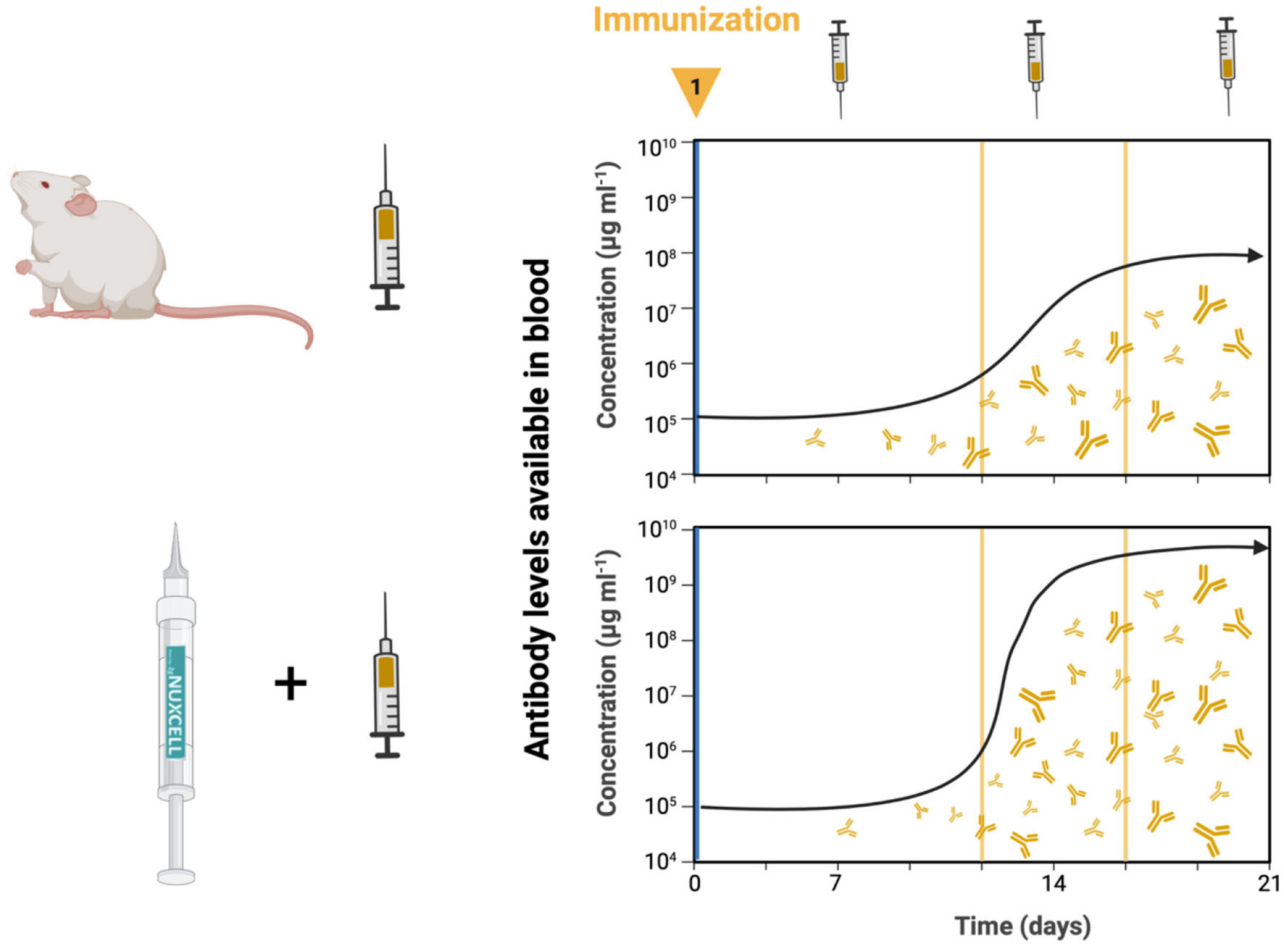
A significant increase occurs at the circulant antibody concentrations in Nuxcell Neo^®^ treatment 14 days post-vaccine and this remains up to at least 21 days; on the other hand, there was no variation in inflammatory evaluation. Nuxcell Neo^®^ enhances vaccine effectiveness.

Considering the parameters analyzed in this study, there was a significant increase in circulating antibodies in the Nuxcell Neo^®^ group, while on the other hand, there was no change in cytokine levels, which is justifiable since there was no presence of insult (Figure 4). Several studies have been designed to evaluate the relationship between immune responses, intestinal microbiota, and vaccine efficiency (7, 9, 31, 34). There is widespread recognition that the gut microbiota can affect the function and development of humoral immunity and vaccine efficacy (35, 36). Our obtained results confirm that the intestinal microbiota affects the effectiveness of the vaccine. The literature has shown that *knockouts* (TLR5), germ-free mice vaccinated against influenza and treated with antibiotics, exhibited low levels of antigen-specific cells, and low IgG concentrations were observed 1 week after vaccination (34). Interestingly, gut microbiota restoration controls the vaccine-specific IgG response (34). Thus, microbial manipulation efforts, especially before 6 months of age, using probiotics and/or altering the diet, can be effective for an optimal vaccine response (37). Our study is in line with the literature and shows that supplementation with Nuxcell Neo^®^ was effective in terms of vaccine response when compared to the group that did not receive supplementation.

For example, infants who received the intramuscular vaccine for tetanus-hepatitis B, oral polio vaccine (OPV), and Bacillus Calmette-Guérin (BCG) vaccine showed detectable levels of *B. longum* and specific T-cell, IgG, and IgA responses to poliomyelitis. In contrast, a higher abundance of Pseudomonadales and Enterobacteriales was associated with lower IgG levels and cellular responses (8, 38). Another study in infants who received BCG, tetanus toxoid, OPV, and hepatitis B corroborates previous results that the abundance of *Bifidobacterium* and *Lactobacillus* in early childhood can enhance the protective effects of vaccines by increasing immunological memory (9). Another previous human study (39) using supplementation with *Lactobacillus rhamnosus* and *Bifidobacterium longum* in children also showed an increased vaccine-specific IgG response. Huda et al. (9) concluded that Bifidobacterium and Lactobacillus colonization at the time of vaccination is associated with sustained vaccine-specific memory T cell and antibody responses at both systemic and mucosal levels.

Finally, *Lactobacillus* and *Saccharomyces* are the strains that provide significant health benefits for both animal and human health, playing a role in metabolic modulation, prevention of infection, and reduction of allergic symptoms (40, 41). Consumption of *Lactobacillus, Saccharomyces*, and *Bifidobacterium* in newborns showed an increase in IFN- γ and secretion cells (42). In addition, when combined, these strains resulted in better antibody responses after Hepatitis B vaccination (39).

## Conclusion

Our results show an increase in vaccine-induced antibody levels in animals treated with Nuxcell Neo^®^ compared to untreated animals, confirming the positive effect of Nuxcell Neo^®^ on the humoral adaptive immune response. Besides this, there are no relevant changes in cytokine levels, which demonstrates that the animal's health remains stable, without oscillations during the 21 days of follow-up in both groups. From the perspective of our study, the relationship between gut microbiota and immune response influenced by probiotic immunomodulation of vaccine efficacy could be explored for other immunization protocols.

## Limitations

It is important to emphasize that Nuxcell Neo^®^ is a multivitamin compound and this composition does not only contain probiotic strains, but it is also necessary to take into account that the formulation was developed with the aim of restoring the intestinal microbiota and increasing the immune response when administered.

## Data availability statement

The raw data supporting the conclusions of this article will be made available by the authors, without undue reservation.

## Ethics statement

The animal study was approved by the Animal Care and Experimentation Committee of UNESC (Protocol 16/2022). The study was conducted in accordance with the local legislation and institutional requirements.

## Author contributions

GJ, NG, MR, DS, RÁ, and MM were responsible for the study design and protocol. NG, RS, MF, EC, MM, and JA performed the experiments. All authors were involved in interpreting the data, wrote the manuscript, commented and approved the final version, and had full access to all data.
